# Modular Psychotherapy Outcomes for Youth With Different Latent Profiles of Irritability and Emotion Dysregulation

**DOI:** 10.3389/fpsyt.2021.618455

**Published:** 2021-04-16

**Authors:** Spencer C. Evans, Melissa A. Wei, Sherelle L. Harmon, John R. Weisz

**Affiliations:** ^1^Department of Psychology, Harvard University, Cambridge, MA, United States; ^2^Department of Psychology, University of Miami, Coral Gables, FL, United States

**Keywords:** irritability, cognitive-behavior therapy, dysregulation, behavioral parent training, emotion regulation, youth psychotherapy, transdiagnostic, modular

## Abstract

**Background:** Severe irritability is a common, impairing problem among youth referred for mental health services, but evidence to guide care is limited. Treatment research can be advanced by adopting a transdiagnostic perspective, leveraging existing evidence-based treatment (EBT) techniques, and situating irritability within the context of emotion dysregulation. Accordingly, this study examined treatment outcomes for youth with different levels of irritability and dysregulation who received cognitive-behavioral therapy (CBT) or behavioral parent training (BPT) in a modular EBT framework.

**Method:** We analyzed data from a community-based implementation trial of a transdiagnostic youth psychotherapy. Two-hundred treatment-referred youths (7-15 years; 47% female; 33% White, 28% Black, 24% Latinx, 14% multiracial, 2% other) and their caregivers completed measures of clinical problems and emotion dysregulation at baseline, with repeated outcomes assessments over 18 months. First, latent profile analysis was applied to baseline irritability and emotion dysregulation data; then, latent growth curve models were used to examine outcome trajectories, controlling for covariates.

**Results:** A two-class solution fit well, differentiating youth with high (*n* = 54) vs. low (*n* = 146) levels of dysregulation and irritability at baseline. Nearly all high-dysregulation youth received either BPT (*n* = 26) or CBT-Depression (*n* = 23). Across measures, both groups showed statistically and clinically significant improvements over time. High-dysregulation youth had greater baseline severity than low-dysregulation youth, but otherwise their longitudinal trajectories were mostly similar, with few between-group slope differences. There was virtually no evidence of differential effects for BPT vs. CBT on clinical outcomes.

**Conclusions:** Youth with severe irritability and dysregulation, treated with a transdiagnostic, modular, EBT approach, showed significant within-person improvements over time. Their outcome trajectories did not differ according to whether they received BPT or CBT. Findings extend the literature on modular, transdiagnostic, and EBT approaches for irritability and dysregulation, suggesting comparable benefits associated with BPT and CBT when treatment selection is guided by comprehensive assessment.

**Clinical Trial Registration:** www.ClinicalTrials.gov, identifier: NCT03153904.

## Introduction

Severe irritability is a common treatment concern among children and adolescents (herein “youth”) referred for mental health services (1). Although some degree of irritability is normative across development, severe irritability is defined as a problem of emotion dysregulation characterized by a heightened proneness toward anger (2–4). A transdiagnostic symptom, irritability is an essential or associated feature of many different diagnostic categories (e.g., disruptive, depressive, anxiety, personality, and stress-related disorders). Despite recent advances in the developmental psychopathology of irritability (5–7), research to guide assessment and treatment remains limited. The best available evidence points to behavioral parent training (BPT) and cognitive-behavioral techniques (CBT) as being effective for irritability (2, 3, 8), and some research suggests these approaches may be *more effective* in a personalized, transdiagnostic format (9). However, CBT and BPT are seldom investigated together in the same study, making it hard to draw conclusions about relative effects. Further, it is challenging to interpret the evidence on treatment of irritability and related constructs [e.g., chronic irritability; severe mood dysregulation; Disruptive Mood Dysregulation Disorder (DMDD); symptoms of Oppositional Defiant Disorder (ODD)] because they have been inconsistently defined and measured in the literature (6), and available studies and treatments have most often focused on related problems like ADHD (10, 11). Responding to these challenges, researchers have emphasized the need to examine irritability from more established conceptual frameworks, including emotion regulation theory (4, 7, 12–14). The current study advances this literature by investigating clinical outcomes among youth with elevated irritability and emotion dysregulation, treated primarily with BPT or CBT in a transdiagnostic, personalized framework.

Broadly, emotion dysregulation refers to a maladaptive pattern of emotional management and expression (15, 16). Theorists have come to view emotion dysregulation as a common feature across many, if not most, forms of psychopathology (16, 17). It has been proposed that emotion dysregulation arises from dysfunctions in the processes that *generate* emotion and occurs due to problematic emotional arousal and reactivity—i.e., when the type, intensity, frequency, and duration of experienced emotions interfere with appropriate goal-directed behavior (18–20). Others have focused on emotion dysregulation that arises from dysfunctions in the processes that *regulate* emotions—i.e., from dysfunctions in individuals' emotional awareness and understanding, emotion regulation goals, and ability to implement different emotion regulation strategies (21–23). These views are not mutually exclusive, and various attempts at synthesis have been made [e.g., (24)]. In the context of youth psychopathology, emotion dysregulation has been characterized (17) by the occurrence emotions that (a) *endure*, despite attempts at regulation; (b) *interfere* with appropriate behavior; (c) are *context-inappropriate*; and (d) *change atypically*, that is, too abruptly or too slowly. These parameters align with current thinking about youth irritability, defined as an “increased proneness to anger compared with peers at same development level,” situated beneath the supraordinate construct of emotion dysregulation (p. 722) (2). Diagnostically, these terms algin with DMDD in DSM-5 (25) and with ODD with Chronic Irritability/Anger in ICD-11 (6), as well as across internalizing and externalizing psychopathology more broadly.

Indeed, the two components of emotion dysregulation—generation and regulation—are involved in the development and maintenance of youth internalizing and externalizing problems. Among youth with anxiety disorders/symptoms, researchers have documented greater intensity and frequency of negative emotional experiences (26); difficulties using cognitive reappraisal effectively (26, 27); and deficits in understanding and managing emotions (28). Youth with internalizing symptoms are also more likely to use emotion regulation strategies that increase negative emotion and functional impairment. Adolescents with anxiety and depressive symptoms show less frequent use of more adaptive strategies like cognitive reappraisal, problem-solving, and acceptance, and more frequent use of maladaptive strategies like avoidance, suppression, and rumination (29). Patterns of emotion regulation strategy use have also been implicated in youth externalizing problems and disorders, with anger rumination predicting aggressive behavior (30–32). High emotional reactivity, deficits in emotional understanding, and difficulty in managing negative emotions have been linked to aggressive behavior among youth both concurrently (33–35) and prospectively (36). And youth with ADHD are more likely to experience intense negative and positive emotions and show deficits in emotion regulation (37, 38). Importantly, emotion regulation strategies are not only a feature or correlate of psychopathology; they also predict increasing psychopathology over time (e.g., rumination and internalizing problems) (39, 40). Given these findings, it seems reasonable that research might be advanced through transdiagnostic approaches that collectively considers these multiple dimensions of emotion (dys)regulation and broad and narrow forms of psychopathology.

Severe irritability represents one form of emotion dysregulation that is implicated across the spectrum of psychopathology (2, 4). Like emotion dysregulation more broadly, youth irritability shows robust associations with anxiety, depressive, and externalizing disorders (41). Nosologically, the chronic form of severe irritability (i.e., not limited to mood episodes) has been situated as a disorder of depressive mood and disruptive behavior (6). Emotion dysregulation and irritability are both viewed as transdiagnostic phenomena (2). Many of the emotion-regulatory deficits that are maladaptive in other areas of psychopathology also play a role in irritability (4, 13). Indeed, the very term “dysregulation” is often used to name dimensions and categories of youth irritability, such as DMDD and SMD (3). The overlap among relevant diagnostic categories and absence of nosological consensus around irritability and dysregulation underscores the need for researchers to use empirical methods—and to evaluate these methods—for identifying severely irritable, dysregulated youth in clinical research. Accordingly, the present study seeks to advance the literature by considering multi-informant indicators of irritability and emotion dysregulation in forming subgroups, empirically derived through latent profile analysis.

One critical gap in the literature concerns the psychosocial treatment of youth irritability and dysregulation. The last half-century of psychotherapy research has seen considerable growth in the number of treatment protocols for psychopathology, most of which target rather specific problems or diagnostic categories (e.g., depression, ADHD) (42). Among existing evidence-based therapies, two have been highlighted as first-line interventions for youth irritability: CBT and BPT. A broad intervention framework, CBT has substantial empirical support for improving symptoms across a variety of youth mental health concerns including anxiety, depression, and aggression—all of which can include irritability. Youth CBT is primarily child-directed and often considered a first-line treatment for youth with emotional disorders. Treatment focuses on teaching youth specific skills for regulating and expressing their emotions. Targeting a different set of mechanisms related to youth psychopathology, BPT is considered the first-line and most effective treatment for children presenting with aggressive or disruptive behavior. Focusing mainly on the youth's caregiver(s) and social environment, BPT seeks to alter parenting practices and reverse the negative parent-youth interactions that reinforce youth disruptive behavior. Core BPT components include labeled praise for appropriate behavior, giving effective directives, ignoring attention-seeking behaviors, and consistent implementation of consequences.

Recent developments in intervention science have increasingly moved away from problem- or disorder-specific protocols and in a more *transdiagnostic* direction (43, 44). This has partly reflected the growing recognition that patient presentations do not usually fit cleanly within a single category like the ones around which manualized therapies have been designed. Presentations of severe irritability and emotion dysregulation have therefore been identified as strong candidates for transdiagnostic youth psychotherapies (45, 46). One approach, the *Modular Approach to Therapy for Children with Anxiety, Depression, Traumatic Stress, or Conduct Problems* (MATCH), is a modular, transdiagnostic intervention targeting multiple forms of psychopathology by bringing together common therapeutic procedures shown to be effective (47). Specifically, MATCH includes behavioral/cognitive-behavioral strategies organized within protocols targeting specific psychological problems, including CBT for anxiety, depression, and trauma, and BPT for conduct problems.

We recently re-analyzed data from a randomized effectiveness trial (48) of MATCH to investigate its effectiveness for youth with severe irritability. Overall, results showed that youth with severe irritability who had been randomly assigned to received MATCH tended to show greater improvements compared to those who had received treatment with standard manualized therapies or usual care (9). Yet, this study was primarily a trial of intervention *format* (i.e., modular/transdiagnostic vs. standard/diagnostic vs. usual care), rendering it challenging to draw conclusions about important questions of intervention *content*—i.e., what techniques work best for irritable, dysregulated youth? The modular transdiagnostic guidance and clinical judgment provides some insight into how MATCH could be used clinically (45), but such guidance must be interpreted with caution in the absence of empirical evidence. More generally, there is a paucity of interventions targeting severe irritability directly (2, 8). More research is needed to understand which approaches and content (parent-focused BPT, youth-focused CBT) might be most effective for this subset of youth.

The present study seeks to help fill these gaps regarding the treatment of severe irritability and emotion dysregulation. Specifically, we use data from a community-based implementation trial of MATCH among 200 youth referred for various emotional and behavioral problems (49). In this sample, the number of youth who received MATCH was more than 3× larger than that analyzed in our previous study (9), allowing for a closer and more sophisticated analysis of outcomes. Thus, in this paper we (a) investigate treatment outcomes for empirically derived classes of youth based on their transdiagnostic profiles of irritability and dysregulation, and (b) test whether clinical outcomes differed according to whether they had received BPT for disruptive behavior or CBT for depressed mood.

## Materials and Methods

### Participants and Procedures

This study was part of a randomized effectiveness trial of MATCH (47), a transdiagnostic, modular, cognitive-behavioral psychotherapy protocol for youth with anxiety, depression, traumatic stress, and/or disruptive behavior [see Weisz et al. (49) for primary study details]. All participating youth received community-based empirically supported psychotherapy via MATCH. Youth and therapists were randomly allocated to either the *Low-Cost* condition (consisting of therapist training in MATCH, plus inexpensive elements like access to online therapist resources) or the *Consultation* + *Low-Cost* Condition (consisting of everything in the Low-Cost Condition plus weekly consultation with MATCH clinical experts). Because there were essentially no differences in clinical outcomes between the two conditions (49), we analyzed the full sample together while accounting for condition as a covariate.

Two-hundred children and adolescents (46% female; *M*_age_ = 10.73 years, *SD* = 2.42, range = 7-15) representing diverse racial/ethnic backgrounds (33% White, 28% Black, 24% Hispanic/Latinx, 14% multiracial, 2% other) and their caregivers were referred for youth therapy at four community outpatient mental health clinics in the Northeastern United States. Study inclusion criteria included ages 6-15 on the day of the initial study telephone screen and scoring in the borderline or clinical range on at least one relevant scale (e.g., Withdrawn/Depressed, Aggressive Behavior, Anxiety Problems, Conduct Problems, Internalizing, and Externalizing) of the Youth Self Report (YSR) or Child Behavior Checklist (CBCL). Youth were excluded if they had a recent (past-year) history of suicide attempts or hospitalization for psychiatric concerns, or if they had been diagnosed with schizophrenia, autism spectrum disorder, or an eating disorder. Families were contacted at 0, 3, 6, 9, 12, and 18 months post-baseline to participate in caregiver-report and youth-report outcomes assessments administered by masked research staff. Informed consent and assent were collected from caregivers and youths, respectively. All study procedures were approved by review boards of Harvard University and the Department of Children and Families for the State of Connecticut.

### Measures

#### Internalizing and Externalizing Problems

Youth internalizing and externalizing problems were assessed using the CBCL and YSR (50). These are widely used, comprehensive rating scales with parallel caregiver-report (CBCL) and youth-report (YSR) forms. Items are rated on a 3-point scale: 0 (*not true*), 1 (*somewhat or sometimes true*), and 2 (*very true or often true*). The CBCL and YSR both generate a Total Problems scale, two broadband syndrome scales (Internalizing Problems and Externalizing Problems), and eight narrowband syndrome scales (e.g., Aggressive Behavior, Anxious/Depressed). These scales have shown strong evidence for internal consistency, reliability, validity, and utility across multiple samples (50). Both measures were administered approximately quarterly from 0 to 18 months. To promote clinical relevance in interpreting our findings, *t*-scores were used for outcomes analyses models using Internalizing, Externalizing, and Total Problem scale data.

#### CBCL/YSR Irritability

Brief parent- and youth-report irritability scales were derived from three items on the CBCL and YSR. These items tap problems with temper loss, mood lability, and stubbornness, rated on the same 0-1-2 scale as described above. The CBCL/YSR irritability scales have been used in several prior studies (51–54). Between the two informants, CBCL irritability has been used more extensively and shows better psychometric properties than YSR irritability, although both were acceptably valid and reliable in a large sample of clinically referred youth (55). In the present investigation, we use these scales as multi-informant dimensional measures of irritability (range: 0-6), administered at all occasions. Baseline irritability data showed that Cronbach's alpha was 0.64 for caregiver-report and 0.63 for youth-report.

#### Top Problems

The Top Problems (TP) scale (56) is an idiographic measure designed for youth and caregivers to separately identify up to three “top problems” of greatest concern to be addressed in treatment. Once youth and caregivers identified their top problems in a pre-treatment interview, they completed weekly and quarterly assessments of the current severity of each problem on a 5-point scale from 0 (*not a problem*) to 4 (*a very big problem*). Given that this is an idiographic measure where top problem content varies across participants, Cronbach's alpha is not an appropriate indicator of reliability. Prior research has shown that the TP has shown strong test-retest reliability, convergent and discriminant validity, and sensitivity to change during treatment (48, 56–58).

#### Irritability Top Problems

One benefit of the TP measure is that the responses given by caregivers and youth can be reliably recoded into their nearest-matching item on the CBCL/YSR using a well-established coding protocol (59, 60). Applying this protocol, we coded which TPs represented at least one of the CBCL/YSR irritability items—that is, whether or not they identified irritability was one of their TPs for treatment at baseline. Youth and caregivers who reported a TP related to temper loss, mood lability, and so on, were identified by this variable as having an irritability TP (1 = present, 0 = absent). This approach has previously demonstrated evidence of validity and reliability (1). Based on double-coding of a randomly selected 49 cases, reliability was excellent for identifying irritability TPs identified by caregiver-report (κ = 0.95) and youth-report (κ = 0.98).

#### Emotion Regulation and Dysregulation

The ***Emotion Regulation Checklist*** (ERC) (61) is a 24-item parent-report questionnaire used to assess youth's ability to manage emotions. Caregivers were asked to rate items on a 4-point Likert scale from 1 (*never*) to 4 (*always*) across two scales: *Emotion Regulation* (e.g., happiness, recovering from negative mood, positive responses to adults and peers) and *Lability/Negativity* (e.g., outbursts of anger, intrusive enthusiasm, frustration, mood swings). Evidence supports the reliability and validity of the ERC (61). In this sample, reliabilities were 0.83 for negative lability and 0.55 for regulation.

The ***Children's Emotion Management Scale*** (CEMS) (62–64) was used to examine how youth managed their *sadness* (12 items), *anger* (11 items), and *worry* (13 items). The CEMS subscales assess youths' *inhibition, dysregulation*, and *coping* patterns with respect to each particular emotion (i.e., 3 subscales, 3-5 items each, within each emotion). Dysregulation measures inappropriate emotional expression (e.g., “I do things like slam doors when I'm mad,” “I cry and carry on when I'm sad.”) and coping measures adaptive methods of emotion regulation (e.g., “When I am mad, I can control my temper,” “I keep myself from losing control of my worried feelings”). Youth and their caregivers were asked to rate items on a 3-point Likert scale from 1 (*hardly ever*) to 3 (*often*). The present analyses used composite scores for *emotion coping* (calculated as the mean of sadness coping, anger coping, and worry coping scores) and *emotion dysregulation* (calculated as the mean of sadness dysregulation, anger dysregulation, and worry dysregulation scores). The CEMS has shown good reliability and validity (2–6), with alpha of 0.71 for coping and 0.64 for dysregulation.

### Analytic Approach

Analyses were conducted within a latent multivariate framework, in two phases.

#### Phase 1: Latent Profile Analysis (LPA)

First, we used latent profile analysis (LPA) to differentiate high- vs. low-dysregulation classes of youth based on 10 indicators: (a) *irritability levels*, as rated on the CBCL and YSR three-item scales; (b) caregiver and youth identification of *irritability as a treatment concern* on the TP measure; (c) *emotion regulation*, as indicated by the CEMS Coping scale and ERC Regulation scale; (d) *emotion dysregulation*, as indicated by the CEMS Dysregulation scale and the ERC Lability/Negativity scale; and (e) *overall psychopathology* on the CBCL and YSR Total Problem raw scores, minus the three irritability items. Irritability TPs were binary variables, treated as probability estimates. Continuous variables were standardized. The emotion regulation variables (CEMS Coping and ERC Regulation) were the only measures where higher scores are considered more favorable; the other measures follow the reverse pattern, where higher scores are considered more severe. As shown in the results, LPA can accommodate these different types and directionality in the data. These 10 variables were selected to collectively capture the key facets of the relevant phenomena—including severe irritability specifically as well as the generation, regulation, and dysregulation of negative emotions broadly—per two informants, multiple methods, and in multiple directions.

Considering the overall complexity of our analytic plan and our *a priori* goal of investigating treatment outcomes for youth with high vs. low levels of multivariate dysregulation/ irritability, we decided to simply estimate a two-class LPA solution and then evaluate its fit overall and relative to a one-class solution. This focused two-class strategy follows in the tradition of some of the earliest applications of latent class/profile modeling (65). More recently, it has been used by Young (66) and Youngstrom (67) to delineate impulsive/reactive aggression constructs in clinical samples. In other LCA/LPA applications, investigators may enumerate many more classes to identify the best-fitting solution—e.g., going up to 6, 7, or more classes, or until convergence problems or fit decrements are encountered. We have adopted this type of thorough k-class enumeration approach in our own work, as appropriate to the research question and the data (68). But this practice requires very large samples and some rather subjective-decision-making on the part of the analyst, leading quantitative experts to recommend that it is almost always advisable to specify a focused *a priori* hypothesis, especially in smaller samples (66). It is possible that our data would reveal that a 3- or 4- or 5-class solution might also fit these baseline data, but such a solution would be inconsistent with the literature and our research question, adding greater complexity to our models while also limiting the utility and generalizability of our findings. Indeed, to continue with class enumeration risks the possibility of spuriously over-extracting classes that do not really exist (69), with the size classes getting smaller, chipping away at the major classes, and threatening the generalizability of the findings. Thus, for this analysis, the two-class solution of high and low dysregulation was well-justified and offered the greatest power for exploring treatment outcomes.

After estimating and evaluating our LPA, all 200 youths were assigned to their most likely class membership: high dysregulation and irritability (hereafter “HIDYS”) or low dysregulation and irritability (“LODYS”). These class assignments and their uncertainty (i.e., posterior probability of class assignment) were exported for subsequent analysis. Characteristics of youth within each latent profile were explored to assess the groups' validity and clinical, demographic, and study characteristics.

#### Phase 2: Latent Growth Curve (LGC) Models

Next, the two LPA-derived classes were specified as predictors of clinical outcome trajectories via latent growth curve (LGC) models. Our overall approach to these analyses draws from the log-transformed modeling strategy used in several prior randomized trial studies (9, 48, 57), including the primary outcomes of the present study (49). That is, we estimated outcome trajectories as longitudinal models wherein our metric of time was the natural logarithm of the number of days since baseline +1. This approach produces a single log-linear slope coefficient useful for interpretation of clinical outcomes. It is also more parsimonious than alternative approaches such as polynomial strategies to achieve a similar result with more terms (linear + quadratic) or estimating different outcome occasions separately (3-month, 6-month, etc., producing 5× more outcomes to interpret). Results confirmed that log-linear slopes fit the data well.

Substantively, our first question in these models was whether HIDYS and LODYS youth differed in their trajectories of improvement over time. It was expected that youth in the HIDYS group would show greater severity *at baseline*, but it was not clear whether they would improve faster or slower than the LODYS group over time. If the HIDYS group improved faster, this might suggest they are showing a greater response to intervention and/or a pattern of regression to the mean (i.e., higher scores have more room to decline simply as a function of chance and time). If the HIDYS group improved more slowly (or showed no change, or even deteriorated), this would indicate that highly dysregulated and irritable youth are not responding as well to MATCH in the same way that the majority of the sample is. And if the two groups showed parallel trajectories of improvement over time, this would suggest MATCH was similarly beneficial for all youth in this sample, irrespective of whether they are HIDYS or LODYS.

Our second question in these LGC models was about the relative effects of MATCH primary problem/protocol area, DEP or CON. That is, based on results of the baseline clinical assessment, youths were classified into their most appropriate treatment track, including (a) those who were viewed as having a primary depression problem and treated with the MATCH *Depression* CBT protocol (“DEP”); and (b) those who were viewed as having a primary disruptive behavior problem, and treated with the MATCH *Conduct Problems* BPT protocol (“CON”). If assignment to DEP vs. CON showed significantly different effects on slopes, this would indicate a differential treatment response favoring either CON or DEP. If they were not different, this would suggest that CON and DEP are similarly effective in for treating youth with HIDYS and LODYS profiles.

These questions were investigated in a series of multigroup LGC models for each outcome variable. Within each model, results were simultaneously estimated separately for two groups (HIDYS, LODYS) with major protocols—the MATCH Depression and Conduct protocols (DEP, CON)—modeled as fixed effect predictors of intercepts and slopes within group. The parameters of interest were the coefficients for latent intercepts and slopes within the HIDYS and LODYS groups, as well as the coefficients for the effects of CON and DEP on those slopes. Study questions were examined through individual model χ^2^ Wald tests of the equality of these coefficients. A statistically significant χ^2^ contrast for the latent intercepts of HIDYS and LODYS would mean one group had higher baseline problem levels than the other group; and a slope difference would mean that one group showed faster problem reduction than the other. Regarding treatment type, if the effect of DEP on slope was different from the effect for CON on slope, this would indicate differential effectiveness such that membership in one protocol/problem group (DEP vs. CON) predicted faster improvement than the other. Significant differences were investigated by examining other model terms to help contextualize the differential effect on slopes, and by probing the differences within each group.

LGC models controlled for covariates representing demographics (age, gender, race), study and treatment variables (clinic, RCT condition, medications, number of sessions), and uncertainty of class assignment. Covariates were specified as predictors of latent slopes and intercepts and were constrained to be equal across groups. Therapist nesting was not included in group-specific models due to the complex patterns of cross-nesting of therapists often treating patients in both LPA groups. Models were estimated in Mplus Version 8 with robust maximum likelihood estimation. Variables were mean-centered within dysregulation groups, such that the latent intercept and slope terms can be interpreted as representing the trajectories for hypothetical average HIDYS and LODYS youth. Baseline indicators of LGC slopes and intercepts were held to variance at 0 for model convergence.

## Results

### Class Differentiation

**Figure 1:**
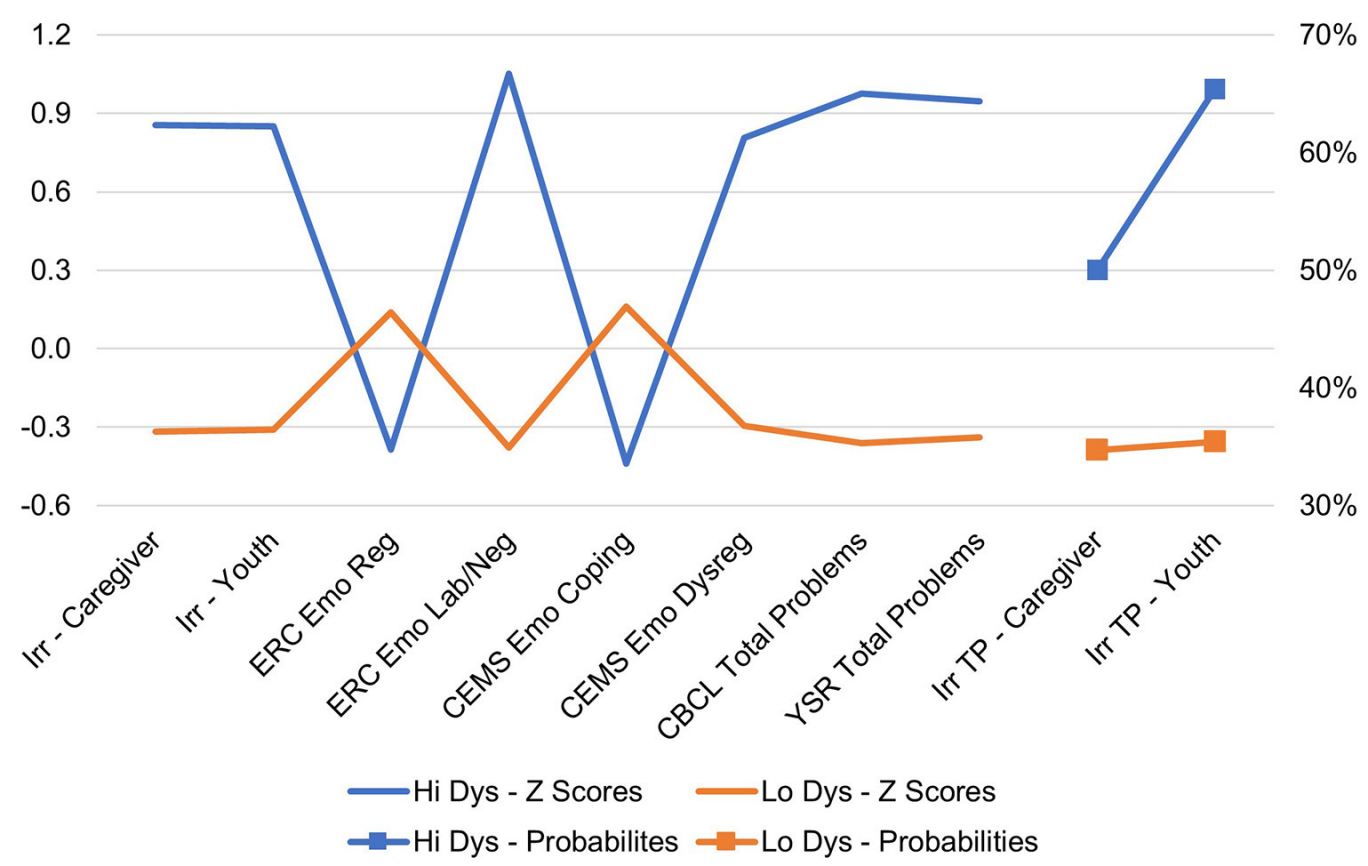
Two latent profiles: high dysregulation (*n* = 54) and low dysregulation (*n* = 146). Irr, irritability; ERC, Emotion Regulation Checklist; CEMS, Children's Emotion Management Scales; CBCL, Child Behavior Checklist; YSR, Youth Self-Report; TP, top problems.

The two-class solution converged successfully and fit the data well. Entropy was 0.839, indicating a high degree of “cleanness” in the separation between the two classes. Average latent class probabilities for most likely class membership was 0.931 for Class 1 (HIDYS) and 0.966 for Class 2 (LODYS). The Lo-Mendell-Rubin (LMR; *p* = 0.0137), Vuong-LMR (VLMR; *p* = 0.0128), and bootstrapped (*p* < 0.0001) likelihood ratio tests all showed that the two-class solution fit the data better than a one-class solution, and the pattern of AIC/BIC results between the one- and two-class models supported this conclusion as well. Figure 1 presents the two profiles that characterized youth with high (*n* = 54; 27%) and low (*n* = 146; 73%) levels of dysregulation and irritability. As shown, the highly dysregulated group was nearly 1 *SD* above the sample mean on measures of irritability, dysregulation, and total problems. They also had below-average levels of coping/regulation skills and were considerably more likely to have a TP defined by irritability, especially by youth report.

Table 1 presents further results showing the clinical and demographic characteristics of the two groups. The HIDYS group showed significantly greater severity on the TP and all CBCL/YSR symptom scales and were significantly more likely to be receiving medication and less likely to be receiving the anxiety protocol. However, there were no differences in terms of study condition, gender, race/ethnicity, age, or number of sessions attended. Table 1 also reveals that youth in the HIDYS class tended to be most often assigned to Conduct (48%) and Depression (43%) MATCH protocols, with only five (9%) falling into the Anxiety or Trauma protocols. Youth in the LODYS Class were somewhat evenly distributed across the protocols for Conduct (34%), Depression (39%), and Anxiety/Trauma (27%). In other words, proportions were sufficient to allow us to compare the effects of Conduct (CON) vs. Depression (DEP) protocols within both the LODYS group (*n* = 49 vs. *n* = 57, respectively) and within the HIDYS group (*n* = 26 vs. *n* = 23, respectively). Given the smaller size of those in the Anxiety/Trauma group, an examination of treatment outcomes for this group was not possible. Instead, analyses focused on the effects of CON group membership and DEP group membership as binary predictor variables, with specific implications for results interpretation noted below where applicable.

**Table 1:** Characteristics of the high and low dysregulation groups.

	**Hi dysregulation** **(*n* = 54)**	**Lo dysregulation** **(*n* = 146)**	***t* or χ^2^**	***p***
**Proportions, *n* (%), *χ*^2^**				
CLC condition, *n* (%)	21 (38.9)	78 (53.4)	3.33	0.068
MATCH-depression	23 (42.6)	57 (39.0)	0.21	0.649
MATCH-conduct	26 (48.1)	49 (33.6)	3.58	0.059
MATCH-anxiety/trauma	5 (9.3)	40 (27.4)	7.44	0.006
Receiving medication	24 (44.4)	41 (28.1)	4.81	0.028
Female	24 (44.4)	68 (46.6)	0.07	0.788
White	13 (24.1)	52 (35.6)	2.39	0.122
Black	16 (29.6)	39 (26.7)	0.17	0.682
Hispanic/Latinx	13 (24.1)	35 (24.0)	0.00	0.988
Multiracial	9 (16.7)	18 (12.3)	0.64	0.425
**Levels, *M* (*SD*), *t***				
Age	10.31 (2.25)	10.88 (2.48)	−1.48	0.141
Sessions attended	10.43 (10.33)	10.90 (8.90)	−0.32	0.747
**Caregiver report**				
TP mean score ^a^	3.72 (0.39)	3.52 (0.51)	2.56	0.011
Internalizing t-score	68.44 (7.51)	63.58 (9.11)	3.51	0.001
Externalizing t-score	72.61 (5.34)	61.07 (8.83)	9.01	<0.001
Total t-score ^a^	72.81 (4.23)	63.90 (6.41)	9.48	<0.001
Irritability sum score ^a^	5.02 (1.07)	2.95 (1.64)	8.61	<0.001
Defiance sum score	4.52 (1.30)	2.47 (1.68)	8.11	<0.001
Aggressive t-score	76.85 (8.36)	62.15 (8.31)	11.09	<0.001
Rule-breaking t-score	67.17 (7.01)	60.05 (6.95)	6.41	<0.001
Attention t-score	71.04 (10.03)	60.95 (8.11)	7.31	<0.001
Withdrawn/Dep t-score	68.52 (10.69)	64.53 (9.96)	2.47	0.015
Anxious/Dep t-score	67.85 (9.30)	62.72 (8.88)	3.58	<0.001
Conduct t-score	70.87 (7.41)	61.41 (7.96)	7.60	<0.001
ODD t-score	72.80 (6.30)	60.91 (8.34)	9.51	<0.001
ADHD t-score	70.22 (7.90)	60.42 (7.71)	7.93	<0.001
Anxiety t-score	65.67 (8.31)	61.64 (8.37)	3.03	0.003
Affective t-score	70.30 (7.41)	63.38 (8.77)	5.15	<0.001
**Youth report**				
TP mean score ^a^	3.39 (0.72)	3.15 (0.72)	2.12	0.035
Internalizing t-score	65.34 (9.84)	54.77 (10.62)	6.32	<0.001
Externalizing t-score	64.09 (9.07)	50.44 (8.47)	9.85	<0.001
Total t-score ^a^	67.34 (8.28)	54.19 (9.33)	9.04	<0.001
Irritability sum score ^a^	3.94 (1.55)	1.91 (1.50)	8.31	<0.001
Defiance sum score	3.30 (1.31)	1.75 (1.16)	8.08	<0.001
Aggressive t-score	69.25 (10.71)	54.93 (5.79)	12.02	<0.001
Rule-breaking t-score	57.66 (6.41)	52.88 (3.79)	6.43	<0.001
Attention t-score	69.09 (10.85)	57.12 (7.47)	8.77	<0.001
Withdrawn/Dep t-score	64.70 (10.94)	57.23 (7.38)	5.49	<0.001
Anxious/Dep t-score	65.04 (11.11)	57.23 (7.58)	5.62	<0.001
Conduct t-score	64.58 (9.82)	54.48 (5.51)	9.10	<0.001
ODD t-score	64.45 (7.60)	54.54 (5.26)	10.35	<0.001
ADHD t-score	65.83 (7.67)	56.94 (6.64)	8.00	<0.001
Anxiety t-score	61.06 (8.73)	57.78 (7.53)	2.60	0.010
Affective t-score	65.91 (9.99)	57.32 (7.34)	6.58	<0.001

a *Denotes a variable that was included in the LPA model that differentiated the two classes*.

### Clinical Outcomes for Youth With High and Low Dysregulation

**Figure 2:**
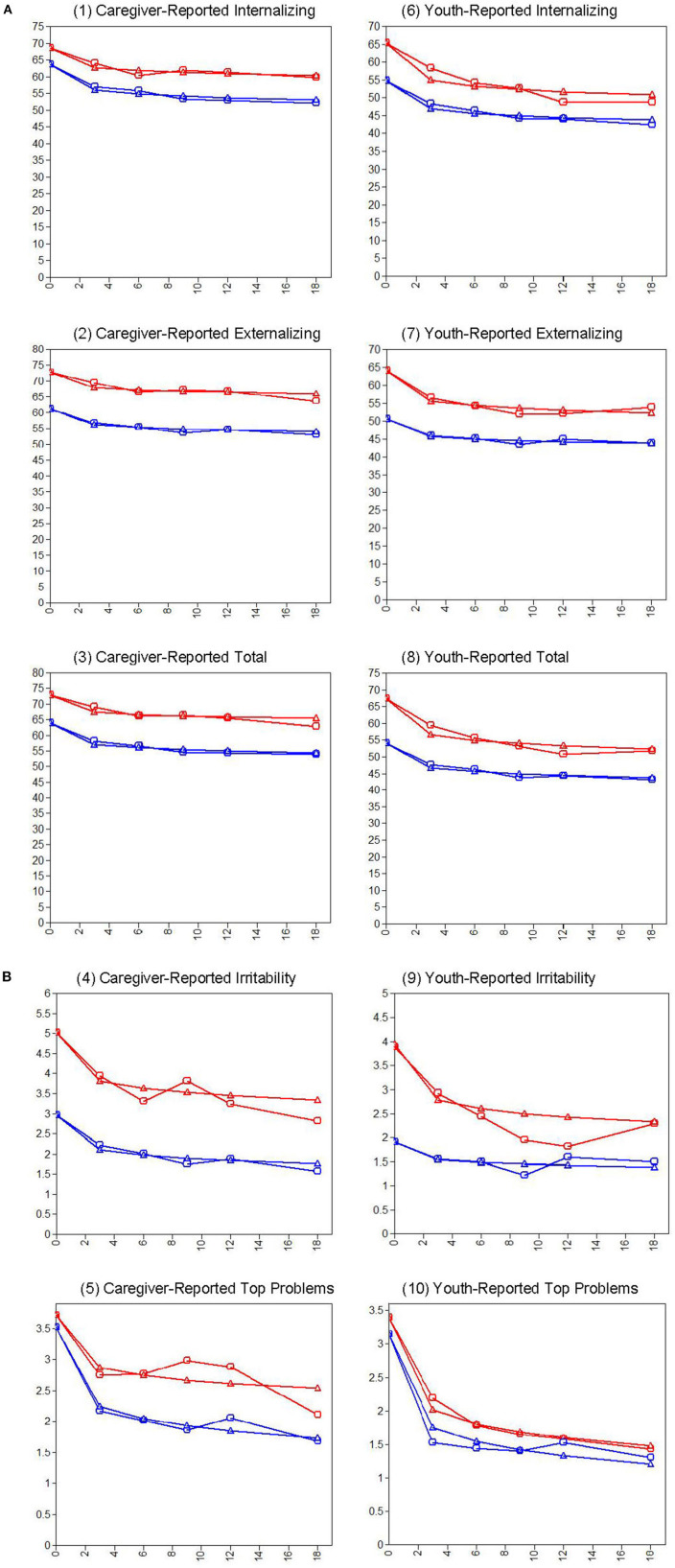
**(A)** CBCL and YSR problem trajectories for youth in the high dysregulation (Red) and low dysregulation (Blue) groups over time (0 to 18 months). Models control for the covariates noted previously (Table 2). **(B)** Irritability and top problem trajectories for youth in the high dysregulation (Red) and low dysregulation (Blue) groups over time (0 to 18 Months). Models control for the covariates noted previously (Table 2).

Outcome trajectories for these the HIDYS and LODYS groups were examined in a series of ten LGC models—five for youth-report variables and five for caregiver-report variables. For brevity and clarity, these results are presented in Tables 2, 3 and Figures 2A,B organized by coefficient and model number. That is, across all table sections and figure panels, results labeled with the same model number (#1-10) were generated from the same LGC model. The model-implied and observed outcome trajectories followed by youths in the HIDYS and LODYS groups are presented in Figures 2A,B, with the corresponding intercept and log-linear growth coefficients reported in Table 2. Generally, the degree to which group intercepts (i.e., baseline levels) and log-linear slopes (i.e., change over time) appear visually similar in these charts is a reasonable indicator of whether they are statistically different, with the exact χ^2^ (*df* = 1) difference tests reported in the far right column of Table 2.

**Table 2:** Latent intercept and log-linear slope growth terms for high and low dysregulation groups.

**LGC model term**	**Hi dysregulation**	**Lo dysregulation**	**Hi vs. Lo**
**LGC models (#1-10)**	**Est (SE)**	**Est (SE)**	**χ^2^ (*df* = 1)**
**Intercept coefficient**			
Caregiver internalizing	68.44(0.80)^***^	63.58(0.58)^***^	24.00^***^
Caregiver externalizing	72.61(0.57)^***^	61.07(0.56)^***^	209.91^***^
Caregiver total	72.82(0.46)^***^	63.90(0.47)^***^	183.60^***^
Caregiver irritability	5.02(0.13)^***^	2.95(0.12)^***^	133.44^***^
Caregiver top problems	3.72(0.05)^***^	3.52(0.04)^***^	9.18^**^
Youth internalizing	65.34(1.26)^***^	54.77(0.83)^***^	49.12^***^
Youth externalizing	64.09(0.99)^***^	50.44(0.63)^***^	136.09^***^
Youth total	67.34(1.06)^***^	54.19(0.75)^***^	103.43^***^
Youth irritability	3.94(0.18)^***^	1.91(0.11)^***^	89.97^***^
Youth top problems	3.39(0.09)^***^	3.15(0.06)^***^	5.38^*^
**Log-linear slope coefficient**			
Caregiver internalizing	−1.28(0.21)^***^	−1.67(0.12)^***^	2.63
Caregiver externalizing	−1.04(0.18)^***^	−1.10(0.10)^***^	0.09
Caregiver total	−1.18(0.19)^***^	−1.51(0.12)^***^	2.29
Caregiver irritability	−0.27(0.04)^***^	−0.19(0.02)^***^	3.05^+^
Caregiver top problems	−0.19(0.02)^***^	−0.28(0.01)^***^	12.18^***^
Youth internalizing	−2.32(0.31)^***^	−1.75(0.15)^***^	2.64
Youth externalizing	−1.87(0.25)^***^	−1.05(0.12)^***^	9.06^**^
Youth total	−2.37(0.29)^***^	−1.65(0.14)^***^	5.32^*^
Youth irritability	−0.26(0.05)^***^	−0.08(0.02)^***^	11.58^***^
Youth top problems	−0.30(0.03)^***^	−0.31(0.02)^***^	0.01

*Models control for the following covariates, mean-centered: clinic (3 dummy codes for 4 clinics), study condition, medication status, age, gender, ethnicity (White, Black, Latinx), number of sessions, problem focus (dummy codes for CON and DEP, not ANX), and probability of latent profile class membership. Thus, these model terms can be interpreted as characterizing the clinical trajectories followed by the average youths in the Hi Dys and Lo Dys groups. LGC, latent growth curve*.

+ *p < 0.10*,

* *p < 0.05*,

** *p < 0.01*,

*** *p < 0.001*.

**Table 3:** Effects of DEP and CON problem/protocol area on LGC interprets and slopes.

**Regression effect**	**Hi Dys**	**Lo Dys**	**Hi vs. Lo**	**Dep. vs Con**.
**LGC Models (#1-10)**	**Est (SE)**	**Est (SE)**	**χ^2^ (*df* = 1)**	**χ^2^ (*df* = 2)**
**LGC intercept regressed on DEP**				
Caregiver internalizing	1.23(2.05)	3.28(1.36)^*^	0.42	62.64^***^
Caregiver externalizing	1.32(2.91)	4.13(1.71)^*^	0.76	32.24^***^
Caregiver total	1.02(1.40)	2.76(1.32)^*^	0.90	1.09
Caregiver irritability	−0.23(0.46)	0.73(0.31)^*^	3.14^+^	3.58
Caregiver top problems	0.34(0.22)	−0.09(0.11)	3.33^+^	2.13
Youth internalizing	5.53(4.19)	1.83(2.18)	0.65	11.19^**^
Youth externalizing	3.06(3.22)	3.54(1.66)^*^	0.02	9.66^**^
Youth total	4.35(4.10)	2.57(1.95)	0.16	1.98
Youth irritability	1.35(0.57)^*^	0.25(0.30)	3.08^+^	2.03
Youth top problems	0.67(0.39)^+^	0.15(0.16)	1.57	2.15
**LGC intercept regressed on CON**				
Caregiver internalizing	−5.86(2.22)^**^	−7.88(1.62)^***^	6.29	
Caregiver externalizing	3.87(2.62)	10.58(1.55)^***^	5.10^*^	
Caregiver total	0.09(1.16)	2.00(1.28)	1.24	
Caregiver irritability	−0.72(0.46)	0.94(0.35)^**^	8.74^**^	
Caregiver top problems	0.19(0.22)	−0.17(0.11)	2.20	
Youth internalizing	0.79(4.11)	−4.70(2.10)^*^	1.45	
Youth externalizing	9.21(2.68)^**^	6.45(1.90)^**^	0.67	
Youth total	5.41(4.01)	0.04(1.99)	1.45	
Youth irritability	1.06(0.45)^*^	−0.16(0.31)	5.02^*^	
Youth top problems	0.45(0.37)	0.27(0.17)	0.19	
**LGC slope regressed on DEP**				
Caregiver internalizing	−0.09(0.50)	−0.44(0.34)	0.37	8.06^*^
Caregiver externalizing	−0.18(0.38)	−0.56(0.30)^+^	0.69	0.10
Caregiver total	0.27(0.43)	−0.36(0.33)	1.45	4.05
Caregiver irritability	−0.02(0.08)	−0.12(0.05)^*^	1.06	2.15
Caregiver top problems	−0.06(0.08)	0.03(0.04)	0.98	2.64
Youth internalizing	−1.35(0.99)	0.04(0.40)	1.66	3.03
Youth externalizing	−0.70(0.83)	−0.17(0.30)	0.35	0.29
Youth total	−0.52(1.01)	0.02(0.36)	0.24	1.54
Youth irritability	−0.25(0.15)	0.06(0.04)	3.90^*^	4.00
Youth top problems	−0.18(0.10)^+^	−0.03(0.04)	1.93	0.45
**LGC slope regressed on CON**				
Caregiver internalizing	0.43(0.42)	0.40(0.31)	0.00	
Caregiver externalizing	−0.10(0.30)	−0.50(0.28)^+^	1.02	
Caregiver total	0.58(0.33)^+^	0.18(0.29)	0.87	
Caregiver irritability	0.10(0.07)	−0.09(0.06)	4.48^*^	
Caregiver top problems	0.00(0.07)	0.07(0.04)^+^	0.64	
Youth internalizing	−0.35(0.95)	0.44(0.41)	0.58	
Youth externalizing	−0.44(0.75)	−0.09(0.34)	0.17	
Youth total	0.07(0.94)	0.33(0.40)	0.07	
Youth irritability	−0.06(0.15)	0.10(0.05)^*^	0.98	
Youth top problems	−0.13(0.09)	−0.04(0.05)	0.82	

*Models control for the covariates noted previously (Table 2). LGC, latent growth curve; DEP, Depression problem focus, treated with CBT; CON, Conduct problems focus, treated with BPT*.

+ *p < 0.10*,

* *p < 0.05*,

** *p < 0.01*,

*** *p < 0.001*.

On all outcome measures, the HIDYS group showed significantly higher problem scores at baseline compared to the LODYS group (top portion of Table 2). This is as expected given how the classes were formed, but the consistency of this result across internalizing and externalizing problems is notable because classes were not formed based on those scales. In terms of outcomes, caregiver- and youth-rated internalizing, externalizing, total problems, and irritability all showed statistically significant log-linear declines over time, and this effect was largely similar between the high and low dysregulation groups (bottom portion of Table 2). On caregiver-rated internalizing problems, for example (see Table 2), youth in the HIDYS group started at 68.44 and improved over time at a rate of −1.28 points per log-day, whereas the LODYS group started at 63.58 and improved at a rate of −1.67 points per log-day. These trajectories differed in their baseline scores (χ^2^ = 24.00, *p* < 0.001) but not in their rates of change over time (χ^2^ = 2.63, *p* = 0.105).

This same pattern for HIDYS vs. LODYS results held across 6 of the 10 outcome variables. That is, the two groups differed at baseline but showed statistically similar slopes of improvement over time on CBCL irritability, internalizing, externalizing, total problems, and on youth-reported top problems and internalizing problems. The other 4 outcome measures, where slopes differed, can be interpreted as follows. On youth-rated irritability, externalizing, and total problems, the HIDYS group was more severe at baseline and improved faster over time than the LODYS group. The values on the scale metrics suggest that, despite these statically significant slope differences, trajectories of improvement were clinically significant for both groups. For example, in both groups youth-reported Total Problems dropped several points below the cutoffs for the “Borderline” (*t*-score ≥ 60) and “Clinical” (*t*-score ≥ 63) ranges, per Achenbach and Rescorla's interpretive guidelines (50). In fact, this was the case for the outcome trajectories for both groups, per both informants, on all normed outcome measures. Lastly, on caregiver-rated TP severity, the groups showed somewhat more similar ratings at baseline (though still statistically different), and over time the LODYS group improved faster than the HIDYS group. These diverging trajectories are potentially meaningful, result in a ~1-point score gap at 18-months, with the HIDYS falling closer to the 4 (*very big problem)* end of the severity scale and the LODYS group winding up closer to 0 (*not a problem*).

### Effects of BPT-Conduct and CBT-Depression Protocols on Outcome Trajectories

The effects of LGC intercepts and slopes regressed on DEP and CON are presented in Table 3. Again, these DEP and CON variables represent binary dummy codes for 2 of the 3 possible MATCH primary problem/protocol areas; thus, the values presented in Table 3 can be interpreted as regression coefficients summarizing the effects that membership in the DEP or CON group had on “nudging” the LGC intercepts and slopes, relative to an ANX reference group (for which estimates are not presented). The benefit of this approach is that it yields results for the *absolute* effects that DEP and CON have on LGC slopes and intercepts (i.e., whether the effect is different from zero, reported in the HIDYS and LODYS columns) as well as a method for comparing the *relative* size of those effects in the rightmost two columns. Specifically, the model Wald χ^2^-tests in this table show whether the coefficients for those effects (a) differ from one another (“Dep vs. Con” column) and (b) whether they differ between the dysregulation groups (“Hi vs. Lo” column).

**Figure 3:**
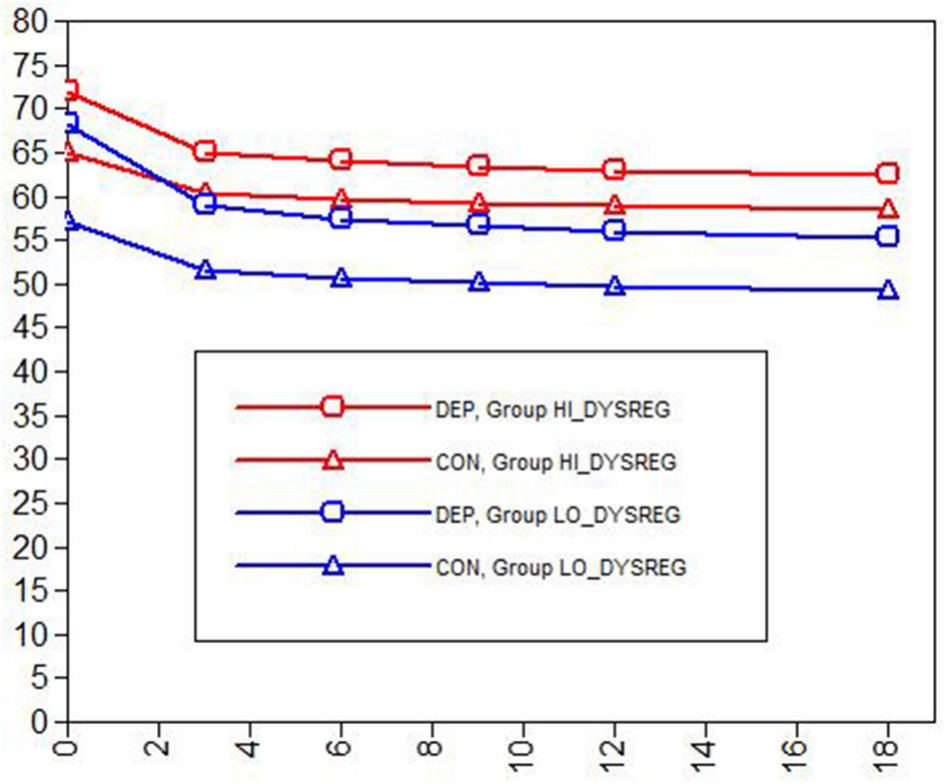
Caregiver-rated internalizing problems by dysregulation group (HI vs. LO) and problem/protocol (CON vs. DEP). DEP, Depression problem focus, treated with CBT; CON, Conduct problems focus, treated with BPT.

Of highest interest are the effects of DEP and CON on latent slopes (see Table 3, far right), where significant slope contrast values served as a gateway for further inspection of the other terms in the table. Here, there was only one outcome out of 10—caregiver-rated internalizing problems—that showed a significant differential effect for DEP vs. CON, χ^2^ (2) = 8.06, *p* = 0.018. When probed, the DEP vs. CON effect on slope was evident in the LODYS group (χ^2^ (1) = 7.37, *p* = 0.007) but not the HIDYS group (χ^2^ (1) = 1.18, *p* = 0.278), and was also accompanied by a differential effect on intercept (χ^2^ (1) = 17.40, *p* < 0.001). These results (see Figure 3) suggest that these effects within the LODYS group might be accounted for by regression to the mean, where those treated with DEP had much higher internalizing *t*-scores at baseline compared to those treated with CON in that same group. It makes sense that youth with higher internalizing scores should be treated with DEP, and that they would decline faster, for reasons of treatment appropriateness and perhaps regression to the mean. Further, the LODYS-CON youth had little room to improve, as they were already below the threshold for clinical significance (*t* < 60) on caregiver-rated internalizing problems at baseline.

## Discussion

We investigated multi-informant clinical outcome trajectories for two latent classes of treatment-referred youth—those characterized by high vs. low profiles of irritability and emotion dysregulation—and we tested whether these trajectories differed for those treated with BPT for conduct problems vs. CBT for depression. Overall, two findings emerged. First, high- vs. low-dysregulation youth were mostly similar in their trajectories, showing statistically and clinically significant improvement over time. Although a few significant differences in slopes emerged (caregiver top problems and some YSR scales), the overall pattern (Table 2, Figures 2A,B) was one in which both groups showed clear improvements across all measures. Second, we found virtually no evidence of different treatment outcomes based on one's primary problem/protocol area. That is, in treating clinically referred youth with (or without) severe dysregulation, a modular transdiagnostic approach involving BPT for externalizing problems and/or CBT for depression appears to be helpful. Importantly, the highly dysregulated youths (27% of the sample) showed significant and comparable improvements in all outcomes regardless of whether they received BPT (*n* = 26) or CBT (*n* = 23) as a primary approach.

One important caveat should be highlighted here to inform further interpretation of our findings: There was not random assignment to problem/protocol (CBT-Depression vs. BPT-Conduct), so causal explanations cannot be drawn as if this were a randomized trial comparing these two approaches. But while random treatment allocation is appropriate for causal inference regarding effectiveness, the world of routine youth mental health care is one of non-randomized allocation. That is, community clinicians do not randomly select one of two protocols to administer to patients referred to them who meet eligibility criteria. Instead, best practices involve conducting a comprehensive baseline assessment and developing a treatment plan involving EBTs based on the best available evidence, clinician judgment, and family preference. In this regard, our non-randomized comparison of CBT vs. BPT for youths with high and low dysregulation represents more of a real-world comparison and a useful contribution to the literature, highlighting the potential value of prospective randomized trials in the future.

In regard to the four variables where the groups' slopes differed, the pattern of this difference varied by informant and appeared to be largely related to baseline differences. For instance, on youth-rated externalizing, total, and irritability, the HIDYS group improved faster than the LODYS group. This pattern may suggest greater clinical benefits for the HIDYS group; however, it is a small difference with unclear practical significance and likely related to regression to the mean (i.e., youth in the HIDYS group started treatment with higher scores, and therefore had more room to improve even if only due to random chance with the passage of time). More importantly, both groups improved to such an extent that they fell well below the borderline and clinical cutoffs on these measures, consistent with the overall pattern described above. However, a unique pattern was observed for caregiver-rated TP severity, where the groups were equivalent at baseline and the LODYS group improved faster than the HIDYS group. Importantly, this suggests that greater levels of dysregulation/irritability predicted slowed improvement and greater treatment needs in these personalized domains of functional problems, at least per caregiver report. Alternatively, this pattern might be explained by the unique properties of the TP measure, which is designed to pull for high scores from *all* participants at baseline, regardless of clinical severity. That is, although the HIDYS and LODYS groups appeared similarly severe on TPs in a *subjective, idiographic sense* (see intercepts in Figure 2A, Panel 5), we know that the HIDYS group was more severe at baseline in an *objective, nomothetic sense* (see Figure 1, Table 2). Thus, caregiver TP scores for the LODYS group should be interpreted in light of these properties—i.e., there might be some degree of inflation in the LODYS baseline TP scores, and one might expect their scores to drop faster simply as a function of the group's lower overall severity (as seen across all other measures and informants).

### Implications and Limitations

One important aspect of this study's design was that a thorough clinical assessment was conducted at baseline in order to guide treatment according to how each case was conceptualized. Youth whose assessment data indicated a primary mood problem received CBT, whereas those whose primary problem was disruptive behavior received BPT (2, 45). Although assessment is sometimes given limited attention in intervention research, accurate identification of the problem is an essential precondition for effective treatment. Perhaps especially in youth mental health, careful measurement is important and challenging, requiring multi-informant, evidence-based assessment approaches (60, 70, 71). Challenges with assessment and diagnosis of severe irritability are what prompted a groundswell of controversy and research in this area to begin with, and which continue presently (3, 6). Differential diagnosis for youth irritability can involve over a dozen different diagnostic categories—cutting across internalizing, externalizing, neurodevelopmental, and other domains—of which irritability is a core symptom or associated feature. Interventions such as MATCH rely heavily on assessment data for (a) the initial routing of the treatment plan to target a core problem area, and (b) ongoing progress-monitoring for treatment personalization and outcomes evaluation (48, 49). Thus, effective intervention for severe irritability begins with an effective assessment to clarify the presentation and focus of treatment (9, 45).

Treatments like MATCH might be considered part of a “first-generation” of transdiagnostic protocols—what some have begun calling “multi-diagnostic” rather than truly transdiagnostic. Research is emerging on promising new transdiagnostic approaches. One example is FIRST (72–74), which includes cross-cutting evidence-based principles that have been shown to be effective for disruptive behavior, mood, and anxiety problems; thus, a therapist could employ one or more principles tailored to address irritability and dysregulation as it manifests across these different dimensions of psychopathology. Another example is the Unified Protocol for Children and Adolescents (UP) (75), which was originally developed for emotional disorders (i.e., anxiety, depression) and has recently been adapted for irritability/anger as well (46, 76). Rather than compiling a large, complicated menu of treatment elements as MATCH does, these newer interventions focus more on transdiagnostic *principles* that have evidence supporting their effectives across major swaths of youth psychopathology (e.g., emotional disorders, or internalizing and externalizing disorders). It is possible that interventions like FIRST and UP, which do not require the clinician to classify each patient into this or that category, would be more efficient and effective. These are important questions for future research.

Some limitations and strengths should be noted. Limitations include the lack of certain instrumentation that could have shed further light on study questions. Namely, diagnostic data were not collected; nor were there multi-informant or multi-modal assessments of irritability, mood, or emotion dysregulation beyond the caregiver and self-report versions included here. More objective interviewer, behavioral, and physiological data could be helpful in future studies, especially to appropriately measure the emotion generation vs. regulation components of emotion regulation phenomena. However, the present study did help overcome these challenges by using carefully selected indicators in line with an irritability and emotion dysregulation framework, to tease apart empirically derived profiles of high vs. low dysregulation. This represents a methodological improvement over prior secondary analyses of trial data, which have employed observed variables with greater measurement error (9, 10). One additional strength of the present study is the diversity reflected in the participant sample, and the implementation of procedures in routine care settings with community clinicians. By nature, LGC models are exploratory, so generalizability and replicability may be limited. Alternative “manual” approaches to sample splitting are sometimes used, such as using a median split or applying cutoffs on one or more measures. This approach has been used in previous analyses attempting to simulate an randomized trial comparing different therapies among a subsample with irritability and impairment (9). However, these approaches are only as strong as the chosen instruments, informants, and cutoffs, which all have their own limitations. In the present study, such concerns were mitigated by our multivariate two-class LPA, our four community outpatient clinics, and the diverse clinical and demographic characteristics of our sample—all of which help promote generalizability and replicability. Lastly, our data cannot speak to specific treatment elements that might be responsible for the observed clinical gains, or the mechanisms of change underlying those gains. It is likely that well-established therapeutic principles in these EBTs (e.g., behavioral activation, changing environmental contingencies, increasing positive attention, restructuring negative cognitions, exposure, and rehearsal of adaptive behaviors) are likely to play important roles [for a practitioner-oriented discussion, see (45)]. It is important for future research to disentangle therapeutic components and mechanisms, to support the development of more personalized and effective approaches.

### Conclusions

The present findings lend support to the notion of applying “old” treatments (CBT, BPT) to “new” problems (irritability/dysregulation), at least when doing so is guided by assessment data and clinician judgment. Well-established cognitive-behavioral treatments and principles provide large toolbox of potentially effective tools. These tools seem to remain effective for practitioners who continue to treat common presentations of emotional and behavioral disturbance in youth, even as researchers work to shed light on new questions about irritability and dysregulation within the context of those presentations. Indeed, evidence-based practice requires using strategies that are known to be effective in general, applying them with a particular youth, a particular clinician, and a feedback loop involving treatment guided by assessment guided by treatment—and so on (77). In this regard, the present study advances the literature while also highlighting important directions for future research. Specifically, there is a need to learn how to make new and old interventions *even more effective* for a variety of clinically referred youth populations—including the most irritable and dysregulated among them.

## Data Availability Statement

The raw data supporting the conclusions of this article will be made available by the authors, without undue reservation.

## Ethics Statement

The studies involving human participants were reviewed and approved by The Committee on the Use of Human Subjects at Harvard University; and the State of Connecticut, Department of Children and Families' Institutional Review Board (IRB). Written informed consent to participate in this study was provided by the participants' legal guardian/next of kin.

## Author Contributions

SE was responsible for the initial conceptualization, data curation, analysis, writing, and editing of this paper. MW and SH contributed to the literature review and initial drafting. JW was principal investigator on the study and made contributions including data, resources, and editing/revising. All authors contributed to conceptualization, reviewing, approved, and agreed to the submission of this manuscript in its current form.

## Conflict of Interest

JW is a co-author of the treatment protocol (MATCH; Chorpita & Weisz, 2009) used in this study and receives some income from its sales. The remaining authors declare that the research was conducted in the absence of any commercial or financial relationships that could be construed as a potential conflict of interest.
